# Farther Remarks on the Efficacy of Blue Vitriol in the Cure of Dropsy
*See Vol. I. page 266.


**Published:** 1789

**Authors:** William Wright

**Affiliations:** the Royal College of Physicians and Royal Society of Edinburgh


III. *Farther Remarks on the Efficacy of Blue Vitriol in the Cure of Dropfy* *. *Communicated in a Letter to Dr.* Simmons, *F. R. S. by* William Wright, *M. D. F. R. S. and of the Royal College of Phyficians and Royal Society of Edinburgh.*

AGREEABLY to my promife, I now fend you fome farther remarks on the cure of

* See Vol. I. page 266.

certain certain ſpecies of dropſy by blue vitriol, which I hope you will deem worthy of a place in the London Medical Journal.

From the number of fatal accidents that have happened from the uſe of copper veſſels, ſpeculative authors have ſet down all the preparations of that metal as virulent in their effects, and of a deleterious quality; but this depends altogether on the ſubſtances with which the copper is combined. It is indeed certain that verdigris, however formed, and taken into the ſtomach in any conſiderable quantity, deſtroys animal life; but, on the other hand, cuprum ammoniacum* (Pharm. Edinenſis), in proper doſes, has been found efficacious in ſeveral caſes of epilepſy and other ſpaſmodic diſeaſes: and blue vitriol has long ago been found effectual in removing obſtinate agues, and lately very beneficial in phthiſis pulmonalis. The laſt-mentioned preparation I have found not only a ſafe but ſucceſsful remedy in certain ſpecies of dropſy, even in aſcites, where there was a fluctuation to be felt in the abdomen, depending perhaps ſolely on a relaxation and debility of the whole ſyſtem. As farther proofs of its good effects in affections

* Copper combined with the volatile alkali.

of this ſort, I ſhall relate the two following caſes:

### CASE I.

John M'Laurin, aged fourteen years, ſon of a poor woman in the town of Falmouth, on the north ſide of Jamaica, from living by the ſide of a moraſs, had contracted an intermittent, which laſted from Auguſt, 1784, till April, 1785, when it firſt degenerated into a remitting, and then into a continued fever. He was reſcued at length from this dangerous ſtate by the ſkill and humanity of Dr. Brown: but after this fever had left him he neither had appetite nor recovered his ſtrength.

When I viſited him about the middle of April he was very weak; his face was pale and bloated; his feet ſwelled towards bed time; and his urine was ſcanty and high coloured.

From the duration of theſe fevers I was at firſt led to think that the hydropic ſymptoms were owing to viſceral obſtructions: I therefore ordered one grain of calomel and twenty drops of laudanum to be given at going to bed. Theſe medicines were taken regularly for the ſpace of a week, but without ſucceſs: for the anafarca became general; the fcrotum and penis were greatly diftended; the abdomen was fwelled, and there was a fluctuation of water in it to be felt.

I now began to think that the opinion I had at firft entertained of the caufe of the fymptoms might not be well founded, and that what I had afcribed to vifceral obftructions might perhaps be merely the confequence of debility: I therefore determined to vary the mode of treatment, and to make trial of the blue vitriol, according to the following formula:

℞. Vitriol. Roman.

Opii ana gr. fs.

Corticis canellæ aromaticæ gr. j.

Mucilaginis Gummi Arabici q. f. f. pilula.

He took this pill morning and evening, and at the end of a few days the dofe of the blue vitriol was increafed to one grain.

This medicine gave him no difturbance. The quantity of urine was remarkably increafed daily. The fwelling foon fubfided; his appetite returned; and in the beginning of May his diforder was quite gone.

CASE

### CASE II.

A woman, named Penny, aged thirty years, who had in general been healthy, had for ſome months an obſtruction of the menſes, for which ſhe had taken a variety of medicines.

In May, 1785, her abdomen was obſerved to ſwell, and, as there was an evident fluctuation of water, different diuretics were adminiſtered, but without ſucceſs; ſo that Dr. Carlyle, who attended her, ſaw there was a neceſſity of tapping her, and this was accordingly done in the beginning of June.

To prevent a return of the dropſy, I recommended the uſe of the blue vitriol and opium. Dr. Carlyle gave her a pill containing, at firſt, one grain, and afterwards two grains of blue vitriol, with one grain of opium, every night at bed time. This medicine ſat eaſy on her ſtomach, and excited no ſort of uneaſineſs in the bowels. The quantity of urine was ſoon remarkably increaſed, and ſhe found herſelf conſiderably mended.

About the middle of June, there being no appearance of the aſcites, Dr. Carlyle pronounced his patient out of danger, and the pills were diſcontinued. The woman recovered her wonted health; the monthly difcharge returned, and fhe has fince continued well.

*Edinburgh,*
March 1, 1789.

